# What reasons do final-year medical students give for choosing the hospitals for their clinical training phases? A quantitative content analysis

**DOI:** 10.3205/zma001246

**Published:** 2019-08-15

**Authors:** Angelika Homberg, Elisabeth Narciß, Katrin Schüttpelz-Brauns

**Affiliations:** 1 University Medicine Mannheim, Medical Faculty Mannheim at Heidelberg University, Department of Undergraduate Education and Educational Development, Mannheim, Germany

**Keywords:** internship, clinical clerkships, career choice, final year [medical education]

## Abstract

**Aim: **In the final (practical) year (PY), students have the opportunity to become familiar with their potential future place of work. At the same time, university hospitals and teaching hospitals compete to recruit the best junior staff from this pool. The aim of this study is to present students' selection criteria for the location of the final year in detail.

**Methods:** On the formal evaluation of the final year at the Mannheim Medical Faculty, students were asked after each training period which reasons led to the selection of the location. Thirty-one subcategories were formed from the free-text responses, sorted according to their controllability and then grouped into 11 main categories. The Mannheim University Hospital introduced an expense allowance in November 2015. The data from the period before and after its introduction, the reasons given for choosing the location for the compulsory and elective subjects, and the reasons given for choosing a teaching hospital or university hospital were evaluated and compared separately.

**Results: **A total of 1,164 questionnaires were evaluated from the period before the introduction of the expense allowance, and 1,120 questionnaires were evaluated from the period after the introduction. Overall, Proximity (19%), Financial incentives (18%) and Subject (18%) were the most frequently cited reasons. The Financial incentives subcategory was the most frequent in period 1 (32%), but lost relevance in period 2 (6%). In contrast, Recommendation gained in importance (from 8% to 15%). A comparison of the lead categories shows that teaching hospitals benefit more from their public image and university hospitals more from the subjects they offer.

**Conclusion:** Students primarily choose the location for their final year for pragmatic reasons, such as Incentives and Living environment, but the Public image of the hospitals also plays a significant role. The frequency of the reasons given differs between compulsory and elective subjects, as well as between university hospitals and teaching hospitals. The results can help to improve the attractiveness of the location in a targeted manner and to present a specific image to the public.

## Zusammenfassung

**Ziel: **Studierende haben im Praktischen Jahr (PJ) die Möglichkeit, ihren potentiellen späteren Arbeitsplatz kennenzulernen. Gleichzeitig konkurrieren Universitätsklinika und Lehrkrankenhäuser bei der Rekrutierung des passenden Nachwuchses aus diesem Pool. Ziel dieser Studie ist, die Auswahlkriterien der Studierenden für die Einsatzorte im PJ differenziert darzustellen.

**Methodik: **Im Rahmen der regulären schriftlichen PJ-Evaluation an der Medizinischen Fakultät Mannheim wurden PJ-Studierende nach jedem Ausbildungsabschnitt gefragt, welche Gründe zur Wahl des Einsatzortes geführt haben. Aus den Freitextantworten wurden 31 Subkategorien gebildet, hinsichtlich ihrer Beeinflussbarkeit geordnet und zu elf Leitkategorien zusammengefasst. Am Universitätsklinikum Mannheim wurde im November 2015 die Zahlung einer Aufwandsentschädigung eingeführt. Die Daten aus der Zeitspanne vor und nach Einführung der Aufwandsentschädigungen, die genannten Gründe für die Wahl des Einsatzortes für die Pflicht- und Wahlfächer sowie für die Einsatzorte Lehrkrankenhaus und Universitätsklinikum wurden jeweils getrennt ausgewertet und verglichen.

**Ergebnisse:** Aus der Zeitspanne vor Einführung der Aufwandsentschädigung wurden 1.164 Fragebögen und aus der Zeitspanne nach Einführung wurden 1.120 Fragebögen ausgewertet. Insgesamt wurden Nähe (19%), Finanzielle Anreize (18%) und Fach (18%) am häufigsten als Gründe genannt. Die Subkategorie Finanzielle Anreize war in Zeitspanne 1 am häufigsten (32%), verlor aber in Zeitspanne 2 (6%) an Relevanz. Hingegen konnten Empfehlungen an Bedeutung (von 8% auf 15%) gewinnen. Beim Vergleich der Leitkategorien ist ersichtlich, dass Lehrkrankenhäuser stärker von der Außendarstellung und Universitätskliniken stärker von dem jeweils angebotenen Fach profitieren.

**Schlussfolgerung: **Studierende wählen den Einsatzort in erster Linie nach pragmatischen Gründen wie Anreize und Lebensumfeld aus, aber auch die Außendarstellung der Einsatzorte spielt eine erhebliche Rolle. Die Häufigkeit der angegebenen Gründe unterscheiden sich sowohl zwischen Pflicht- und Wahlfächern als auch zwischen Universitätsklinikum und Lehrkrankenhäusern. Die Ergebnisse können Kliniken dabei unterstützen, die Attraktivität des Einsatzortes gezielt zu verbessern und nach außen darzustellen.

## 1. Background

In Germany, the final (practical) year (PY) usually begins after 10 semesters of undergraduate medical training and represents the last part of the medical program before the final examination. At most medical schools, the 12 months of the PY are divided into three segments lasting 16 weeks each. Students spend one segment in Internal Medicine and one in Surgery. Since the introduction of national mobility during the PY in April 2013 (revised Licensing Regulation for Physicians (AÄAppO) of 17 July 2012), the location and subject can be chosen almost freely. In addition to each university hospital and the teaching hospitals associated with it, it is also possible to complete individual segments of the PY in Germany or abroad (§ 12 in conjunction with §§ 3,4 of the ÄAppO). 

In medical undergraduate training, the PY represents an important link between the acquisition of competence during the course of study and the independent execution of competences as medical expert [1], [2], [3]. The education of students during the PY in Germany is regulated by the ÄAppO as follows: students should "deepen and expand the medical knowledge, skills and abilities acquired during the preceding studies. They should learn to apply them to individual cases of illness" (ÄAppO of June 2002, §3 subsection 4). In contrast to medical education in other countries, such as the US, German students usually choose the location for the PY themselves [4]. For students, the choice of the location for each PY segment can already be an initial introduction to their future employer and an early specialization in a later professional career. 

In addition to training, the PY is understood here to be a phase in which PY students can try out a potential future job and a certain subject area under real conditions [5], [6], [7]. A positive experience with the PY supervisor can also affect the choice of a future specialization [8]. Conversely, university hospitals and academic teaching hospitals are interested in observing PY students in practice over a longer period of time and in recruiting the best as junior staff. Due to the mobility of medical students within Germany, teaching hospitals and university hospital compete nationwide for PY students and, ultimately, for future young professionals. The research report of the German Hospital Society states that around three-quarters of hospitals had problems filling vacancies in medicine at the beginning of 2010 and that 4% of all physician positions in the inpatient sector remain vacant, at smaller hospitals this is even 6% [9]. According to statistics from the German Medical Association, the number of specialists has been rising slightly since 2010, but demand is also rising at the same time as the percentage of doctors over 59 has grown to 18% [10].

University hospitals and teaching hospitals can benefit from knowing the possible motives of students for choosing particular locations so that a location’s advantages can be presented to the public in specific ways or appropriate incentives can be offered.

According to § 3, subsection 4 of the Licensing Regulations, medical faculties have the option of granting financial or non-financial benefits which may not exceed the requirements for trainees according to § 13, subsection 1, number 2 and subsection 2, number 2 of the Federal Training Assistance Act (BAföG). 

Some faculties also offer living allowances or free meals, accommodation, parking, uniforms and even free use of the fitness studio, sauna and swimming pool [11]. We found that there are hardly any studies available for the German-speaking countries showing the extent to which the corresponding incentives influence students' choice of location or what other criteria are central to their choice [12], [13], [14]. In order to better understand the dynamics behind the selection, free-text responses on the regular PY evaluation of the Mannheim Medical Faculty of the University of Heidelberg were evaluated regarding the motives for selecting the desired location.

The following questions will be investigated:

1. Which criteria play a role in students’ selection of locations for the practical year?
2. How are the reasons given for choosing the University Hospital different from those given for choosing a teaching hospital?
3. What are the differences between choosing the location for compulsory and for elective subjects?
4. What influence does the introduction of an expense allowance have on the choice of the location?

## 2. Methods

### 2.1. The Practical Year at the Mannheim Medical Faculty

Since the 2006/2007 winter semester at the Mannheim Medical Faculty of the University of Heidelberg, medical undergraduate training is provided as a reformed curriculum. The training is based on the MaReCuM (Mannheim Reformed Curriculum for Medicine and Medical Professions). A special feature of MaReCuM is the quartered PY curriculum, which is divided into four 12-week segments in which students focus on Internal Medicine, Surgery, Outpatient Medicine and an elective subject. These subjects can be taken at the University Hospital or at one of the ten academic teaching hospitals. The teaching hospitals are in close contact with the faculty and meet the same educational standards. 

The University Hospital and the teaching hospitals differ in the amount of expense allowance received by PY students. Before October 2015 the Mannheim University Hospital did not offer any expense allowance, but did provide a fixed lunch allowance and work garments free of charge. In November 2015 a monthly expense allowance of 200 Euros was introduced. Most teaching hospitals affiliated with the Mannheim Medical Faculty already offered comparable expense allowances, plus benefits such as clothing and lunch, prior to October 2015. The introduction of the expense allowance at Mannheim University Hospital has had a decisive effect on the applicant behavior of PY students when choosing a hospital for clinical training. Previously, the lack of financial incentives had intensified the search for alternative offers [13].

#### 2.2. Data collection

Since August 2012 all PY students at the Mannheim Medical Faculty have been asked by email at the end of each PY segment to evaluate their current location. This email contains a link to the EvaSys online survey and a personal transaction number (TAN) with which students can login to the system. This procedure enables anonymous participation in the evaluation, whereby non-participation can also be identified by the unused TAN numbers. In total, the PY students are reminded twice after each PY segment. The questionnaire comprises general questions about PY training, self-assessments of satisfaction with PY training and questions about the acquisition of medical skills [15].

#### 2.3. Data analysis

This study included data from the cohorts of August 2012 to May 2014 (period 1) and the cohorts of May 2016 to November 2017 (period 2). The time for each cohort corresponds to the beginning of the PY. The data of the PY cohorts for November 2014, May 2015 and November 2015 were deliberately excluded from the study as they were not fully affected by the expense allowance introduced in November 2015. Also, a transition period has to be expected before the corresponding effect is seen. At the time of data analysis, data from PY segments 3 and 4 for the November 2017 cohort were not yet available for period 2. In order to have a comparable population in both time periods, the data from PY segments 3 and 4 for the May 2014 cohort were not included in the study either (see table 1 (Tab. 1)). 

The following questions were evaluated in this study:

- Question with one best answer: “At which location did you complete the PY quarter?”
- Question with one best answer: “In which subject did you complete this PY quarter?”
- Question with yes or no answer: “Was this your desired location?”
- Open-ended question: “What were the reasons you wanted to be trained at this location?”

Thirty-one subcategories were formed from the open-ended responses for time periods 1 and 2 and clustered into 11 main categories, depending on whether these could be institutionally controlled and actively modified by the particular location. The frequency of these reasons was calculated separately for the main categories and subcategories. The reasons for the choice of the locations were compared descriptively at the level of the main categories for compulsory and elective courses, as well as with regard to the location of the University Hospital and the teaching hospitals for both periods. The frequency of the data for the main categories in time period 1 and time period 2 were checked for differences by means of a chi-square test taking the Bonferroni correction into account (adjusted significance level p<0.0045).

## 3. Results

For period 1 (PY cohorts August 2012 - May 2014), 709 questionnaires were available (61% returned); 16 questionnaires were excluded from evaluation due to lack of information on the location, desired location and reasons. Accordingly, n=693 questionnaires from period 1 could be included in the evaluation. For period 2 (PY cohorts May 2016 to November 2017), n=787 questionnaires were available (70% response) and were included in the evaluation (see table 2 (Tab. 2)). In both periods, 78% of the respondents gave reasons for their choice of location in the free-text field. The number of questionnaires with reasons in the first period was 541 with 928 reasons cited; in the second period, 614 questionnaires with 1,027 reasons cited. 

For the comparison of the reasons between the choice of location for the compulsory and elective subjects, only those questionnaires were included for which the location was also the desired location. For the compulsory subjects, only Surgery and Internal Medicine were included but not the compulsory subject Outpatient Medicine in Mannheim. In period 1,193 out of 221 questionnaires could be evaluated for the reasons given (compulsory subjects n=126, elective subjects n=67) and in period 2,325 out of 343 questionnaires for the reasons given (compulsory subjects n=222, elective subjects n=103). 

For the comparison of the reasons between the University Hospital and the teaching hospitals, only the questionnaires with the stated reasons were used and for which the location was also the desired one. In period 1 this was the case in 361 of 425 questionnaires (University Hospital n=161, teaching hospitals n=200) and in period 2 with 381 of 408 questionnaires (University Hospital n=194, teaching hospitals n=187).

### 3.1. Criteria for the selection of the location for PY training

The criteria named cover a wide range of very heterogeneous motivations. All sub-categories and main categories are shown in table 3 (Tab. 3). Here it can be seen that the reasons Public image and Incentives dominate in the choice of location, followed by Living environment and Subject. At the level of the subcategories, the deciding factors for the choice of PY location in the 1,155 questionnaires with cited reasons are, in particular, Proximity to the location (19%), Financial incentives (18%) and the Offered subjects & interest (18%), followed by Recommendation (12%), Good support (11%), Reputation (9%) and Working climate & team (7%). On the other hand, future aspects such as Future place of work (3%) and Future field of specialty (2%) are hardly mentioned explicitly for the choice of location for PY training. A comparison of the number of mentions for all main categories shows that the institutional factors that can be controlled make up the largest share.

#### 3.2. Differences in motivation between university hospitals and teaching hospitals

**Figure 1:**
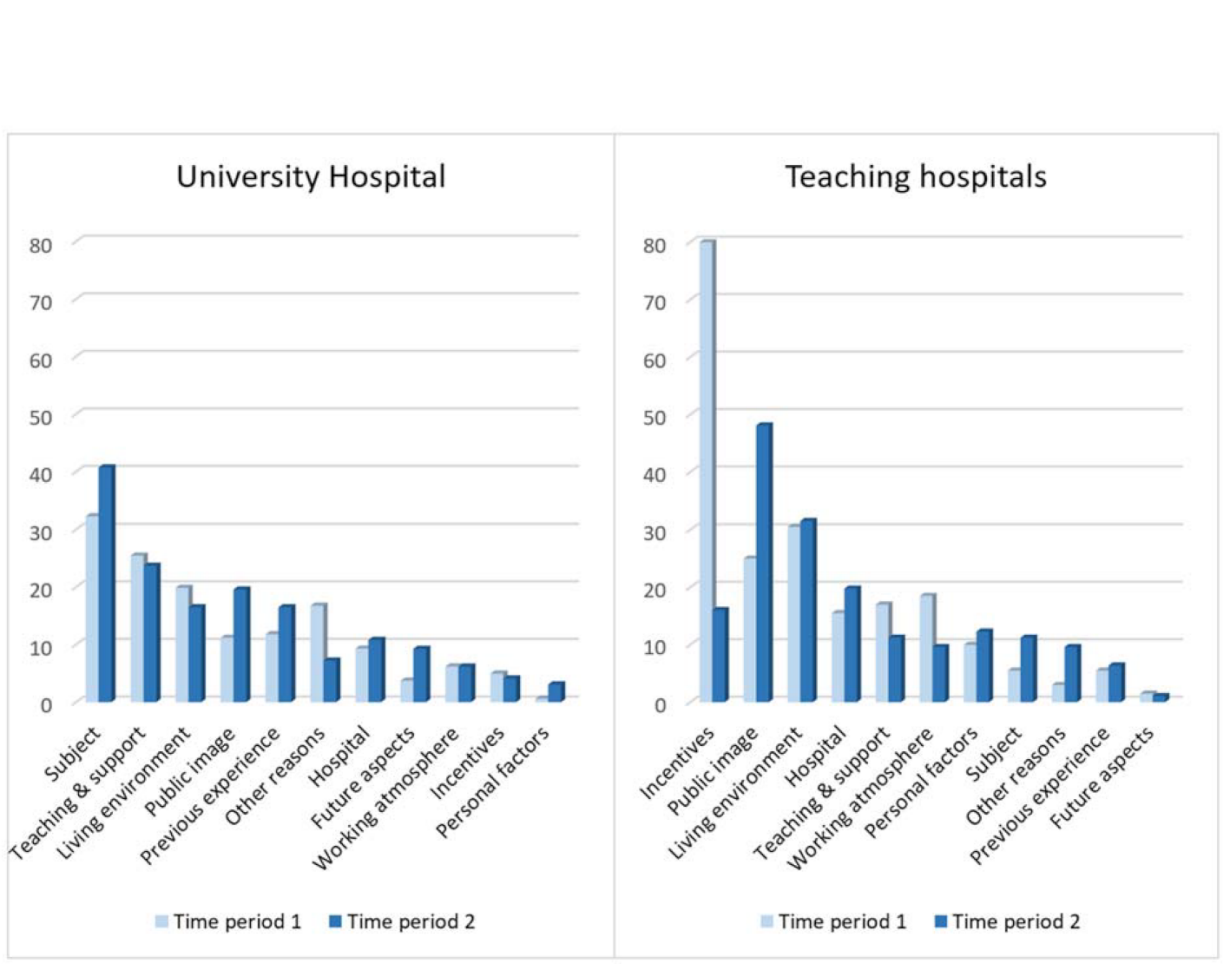
Comparison of the main categories at the Mannheim University Hospital and at the teaching hospitals of the Medical Faculty Mannheim. Percentage of the number of questionnaires with stated reasons and "Yes" for the desired location (University Hospital: time period 1, n=161; time period 2, n=194; teaching hospitals: time period 1, n=200; time period 2, n=187).

Living environment and Public image play an important role in both periods, regardless of the location. In period 1, the teaching hospitals benefit from the lack of Incentives at the University Hospital. Now, that comparable conditions have been created at both locations, it has become obvious that, above all, Subject and Teaching & support are decisive for choosing the University Hospital. Compared to university hospitals, teaching hospitals can benefit more from Hospital size (see figure 1 (Fig. 1)). 

In addition to comparing the main categories (see figure 1 (Fig. 1)), the subcategories were ranked over both time periods for the University Hospital and the teaching hospitals. The most frequently cited reasons are, with reference to 355 questionnaires with a desired location, University Hospital: Subject (26%), Proximity (16%), Doctoral thesis (11%), University & large institution (10%) and Previous experience (10%). In reference to 387 questionnaires with a teaching hospital as the desired location, Financial incentives (36%), Proximity (27%), Recommendation (20%), Small institution (15%), Good support (12%) and Working climate & team (11%) were mentioned most frequently.

#### 3.3. Differences in motivation for choosing of location for compulsory and elective subjects

While PY students are more likely to choose a teaching hospital for the final year in Internal Medicine and Surgery in both periods, the University Hospital is more likely to be chosen for the elective subject (see table 4 (Tab. 4)). 

**Figure 2:**
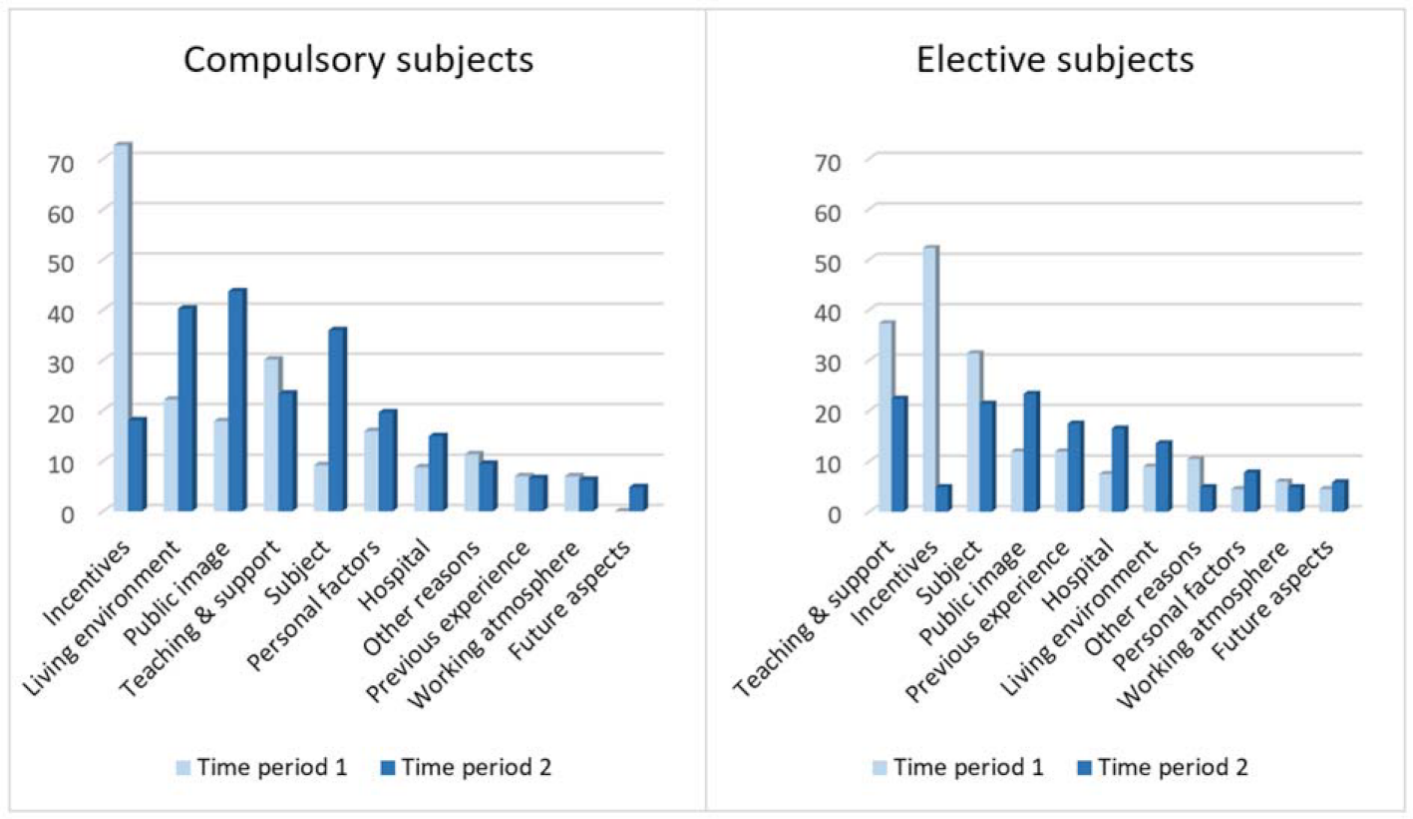
Comparison of the main categories of the hospital for the compulsory subjects (Internal Medicine and Surgery) and for the elective subjects. Percentage of the number of questionnaires with stated reasons and "Yes" for the desired location (compulsory subjects Internal Medicine and Surgery: time period 1, n=126; time period 2, n=222; elective subjects: time period 1, n=67; time period 2, n=103).

The comparison of the main categories is shown in figure 2 (Fig. 2). In the comparison, it is particularly noticeable that Living environment and Public image play a greater role when choosing the location for the compulsory subjects, while Previous experience and Subject play a greater role when choosing the location for the elective subjects. In both areas, Incentives lost relevance in the second period of time, whereas Public image was cited more frequently as the reason for selection. In contrast, for the category Subject the development from time period 1 to time period 2 runs in reverse. While this category gained in importance when choosing the location for the compulsory subjects, the category Subject lost importance when deciding on the location for the elective subject. 

#### 3.4. Influence of the expense allowance on location choice

In period 1, 32% of the reasons mentioned fell under the subcategory Financial ncentives; in period 2 this was only 6%. In period 2, with comparable financial conditions between the University Hospital and the teaching hospitals, the reasons Recommendation (from 8% to 15%), Offered subject & interest (from 16% to 19%) and Ratings (from 2% to 6%) gained in importance. The number of students who can spend their PY at their desired location increased in period 2; the percentage of teaching hospitals increased from 87% in period 1 to 92%, and from 77% to 92% for the Mannheim University Hospital (see table 2 (Tab. 2)). 

**Figure 3:**
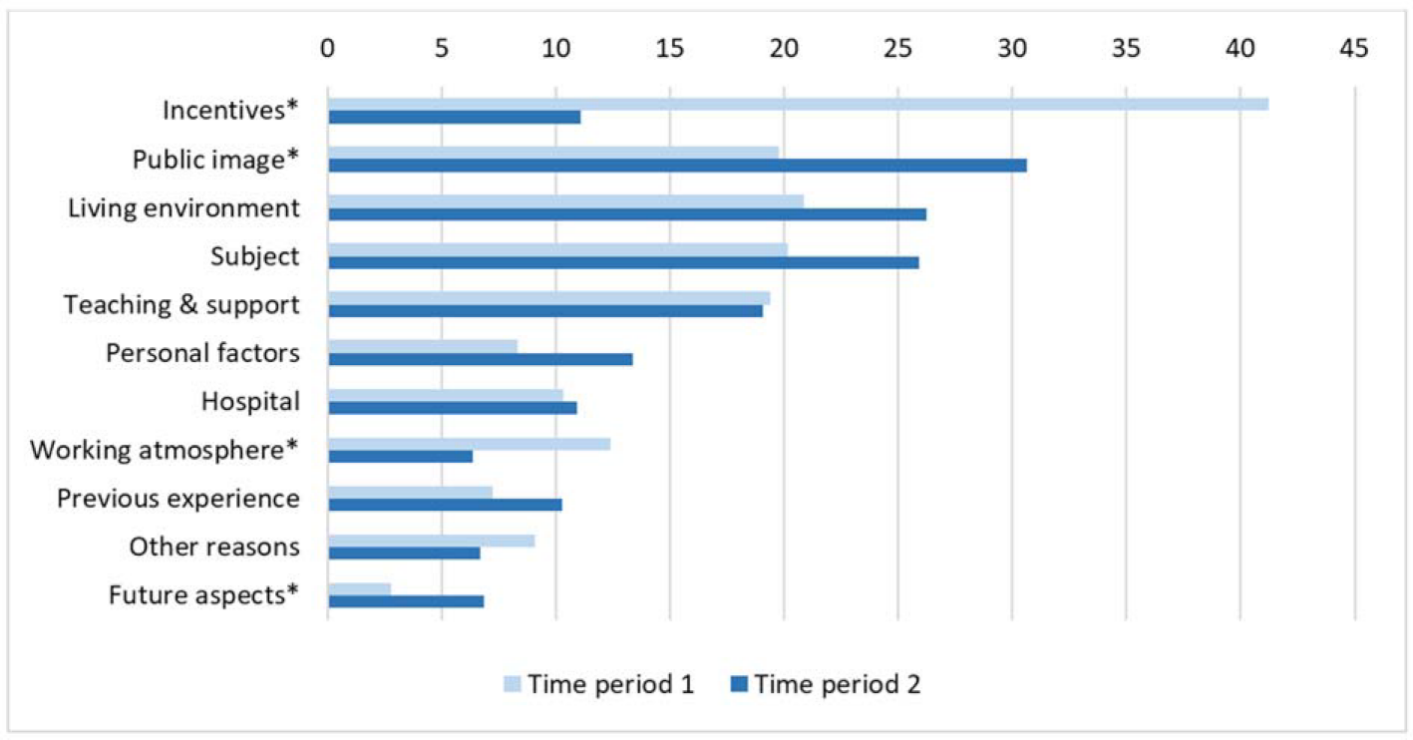
Changes in the main categories from time period 1 to time period 2. Percentage of changes related to the number of questionnaires with stated reasons (time period 1, n=541; time period 2, n=614). *Significant differences between the time periods with p<0.0045 (after Bonferroni alpha adjustment)

The comparison of the main categories' differences between the two time periods was made with the help of chi-square tests, taking the Bonferroni correction into account. This showed that Public image and Future aspects increased significantly in importance in period 2, while Working atmosphere decreased significantly in importance (see figure 3 (Fig. 3)).

## 4. Discussion

Students gave numerous and heterogeneous reasons that demonstrate the individuality of their decision-making process. Nevertheless, decision patterns and trends can be identified from the results. 

The tendency to look for the location for the PY location near the place of residence or the family increases in time period 2 and is indicated much more frequently when choosing a teaching hospital in comparison to the University Hospital. Students who live in or have family in rural areas generally have a longer journey to university hospitals because these are usually located in urban areas. In these cases, teaching hospitals have a clear locational advantage. As part of EY study, 2,000 students in 27 university towns in Germany were asked about their values, goals and perspectives [16]. This study reports that students in Germany focus on family, friends and leisure time, and that the compatibility between family and career is becoming increasingly important. The number of respondents to the EY study who stated that proximity to their place of residence was a very important factor in their choice of future employer was 25% in 2018. The results support the assumption that proximity to one's place of residence will continue to play a significant role in the choice of PY location.

The compulsory PY segments in Surgery and Internal Medicine are much more frequently attended at teaching hospitals. Students often already know the departments at the University Hospital due to their clinical training in prior study years. PY students may therefore consciously choose a teaching hospital in order to get to know a different environment. On the other hand, the University Hospital is used more frequently for the compulsory subject Outpatient Medicine and for the elective subject, since students find a comprehensive range of subjects and a wider spectrum of clinical problems here than at teaching hospitals. Some elective subjects are also offered only at university hospitals leaving no other choice. 

Selection behavior and the reasons for it reflect the fact that most hospitals now pay an expense allowance. As expected, the introduction of the expense allowance at the UMM means that the subcategory Financial incentives, which was particularly relevant when deciding on a teaching hospital, is becoming less important. At the same time, the number of students who say that they spend their PY at the desired location is increasing. It can be assumed that some students decide against their desired location if the corresponding financial incentives are lacking as this may force them to finance their studies by working weekends or night shifts despite the fact they are already completing a full workweek for the PY [17]. According to our results, payment of a standardized expense allowance could lead to more students completing their PY at their desired location and being guided by their professional interests. Whether the payment of an expense allowance also affects intrinsic motivation is not proven. However, it is presumed that PY students will be more motivated during the PY if financial incentives go well beyond basic needs. There are indications that short-term learning goals can be better achieved and that students are more willing to learn in a self-directed manner; however, a long-term impact on performance is questionable [18]. 

Furthermore, it could be shown that in time period 2 Recommendations, in particular by fellow students, and Ratings gain significantly in importance. The extent to which a general trend is emerging cannot be assessed. It is possible that students will increasingly draw on the experiences of fellow students and evaluations like the PY rankings if the general conditions, such as Financial incentives, hardly differ among the different locations. It is interesting to note that the subcategory Reputation remains constant over both periods. Given the same general conditions at all locations, active inquiries will be made among fellow students and acquaintances or ranking lists will be sought in order to make a well-founded decision in favor of a particular location. Thus their experiences have an immediate effect. These experiences are probably based on good teaching and supervision, as well as on the perceived work atmosphere, so that it is assumed that this influence is underestimated by this survey. 

The main categories Subject and Future aspects are also gaining in importance. In these areas, university hospitals can benefit more since they offer a wider range of electives than teaching hospitals and can attract young talent through opportunities for dissertations. 

While the EY study in 2018 identified good job prospects for a career, good earning opportunities and career opportunities, along with personal interest, as the most important motives for choosing a subject of study, these aspects play only a subordinate role in the choice of the PY hospital, since medical students more or less have a job guarantee. Sixty-nine percent of the medical students surveyed in the EY study stated they are sure that after completing their undergraduate training they will quickly find a job that meets their expectations and qualifications [16]. It is therefore possible that students rarely state that the explicit reason for choosing a location is to learn specific skills and abilities in order to have an advantage in terms of future employment in a particular medical discipline. 

It cannot be conclusively assessed to what extent changes in the licensing regulations (revised ÄAppO of 17 July 2012), such as administering the M2 examination earlier and allowing mobility of students within Germany, have contributed to the changes in selection behavior. 

In addition, the question arises as to what extent students are motivated and are in a position to select the location with a view to become specifically prepared for future medical work. The fact that students here are more strongly guided by Public image and Living environment shows once again that it is incumbent on the medical schools to keep an eye on the academic quality of PY clinical training [19], [20].

### 4.1. Limitations

The strength of this study lies in the fact that, when collecting the data, no response possibilities were given for stating the reasons for the choice of PY location. This made it possible to map the whole spectrum of motivations. However, it must be said that in Mannheim this is a reformed curriculum that has implemented a four-part PY. The authors assume that the reasons for the choice of PY location differ only marginally from other universities with regular study programs. A further limitation of the study is the retrospective evaluation of data. It can be assumed that differences between the two time periods are subject to other influencing factors in addition to the above-mentioned introduction of an expense allowance.

## 5. Conclusion

University hospitals and teaching hospitals can present their location features to the public. The following factors play an important role:

- Size and, if applicable, special features of the hospital
- Range of treatments
- (Elective) Subjects
- Possibility of future employment
- Attractiveness of the surroundings and leisure opportunities

The attractiveness of a location can be increased by the following:

- Financial incentives in the form of compensation for expenses, free food, etc.
- Good supervision of interns, trainees and PY students
- Good working atmosphere and respectful interaction on teams

Students’ intrinsic motivation can be promoted if a specific subject or hospital has been represented positively in previous study modules. Teaching hospitals are at a disadvantage, as they are mainly only able to present themselves positively in clinical electives.

## Acknowledgement

We would like to thank Julia Thiesbonenkamp-Maag for her support in creating the main categories and subcategories.

## Competing interests

The authors declare that they have no competing interests. 

## Figures and Tables

**Table 1:**
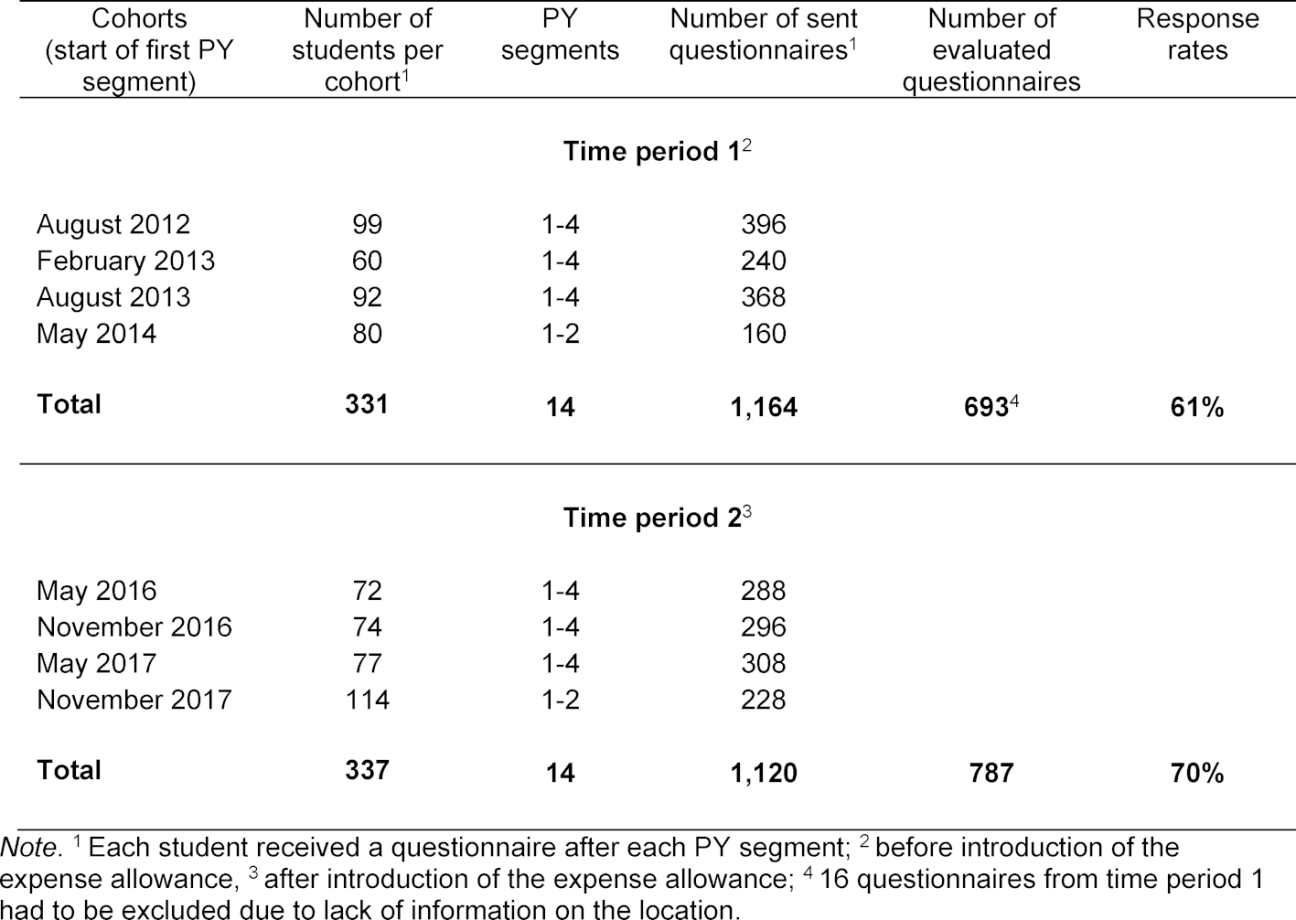
Survey periods and response rates for the time periods before and after introduction of the expense allowance at Mannheim University Hospital

**Table 2:**
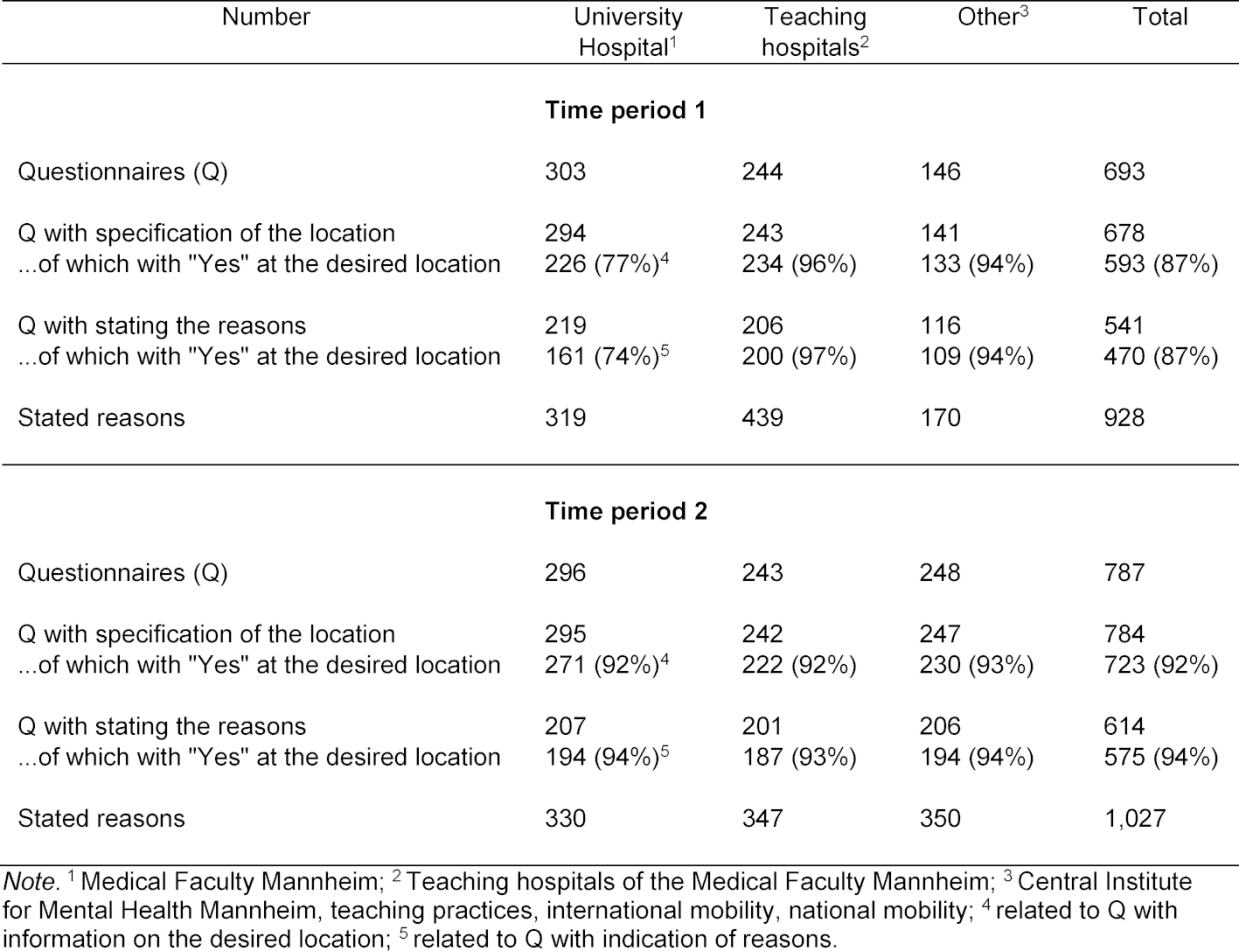
Number of questionnaires and reasons given

**Table 3:**
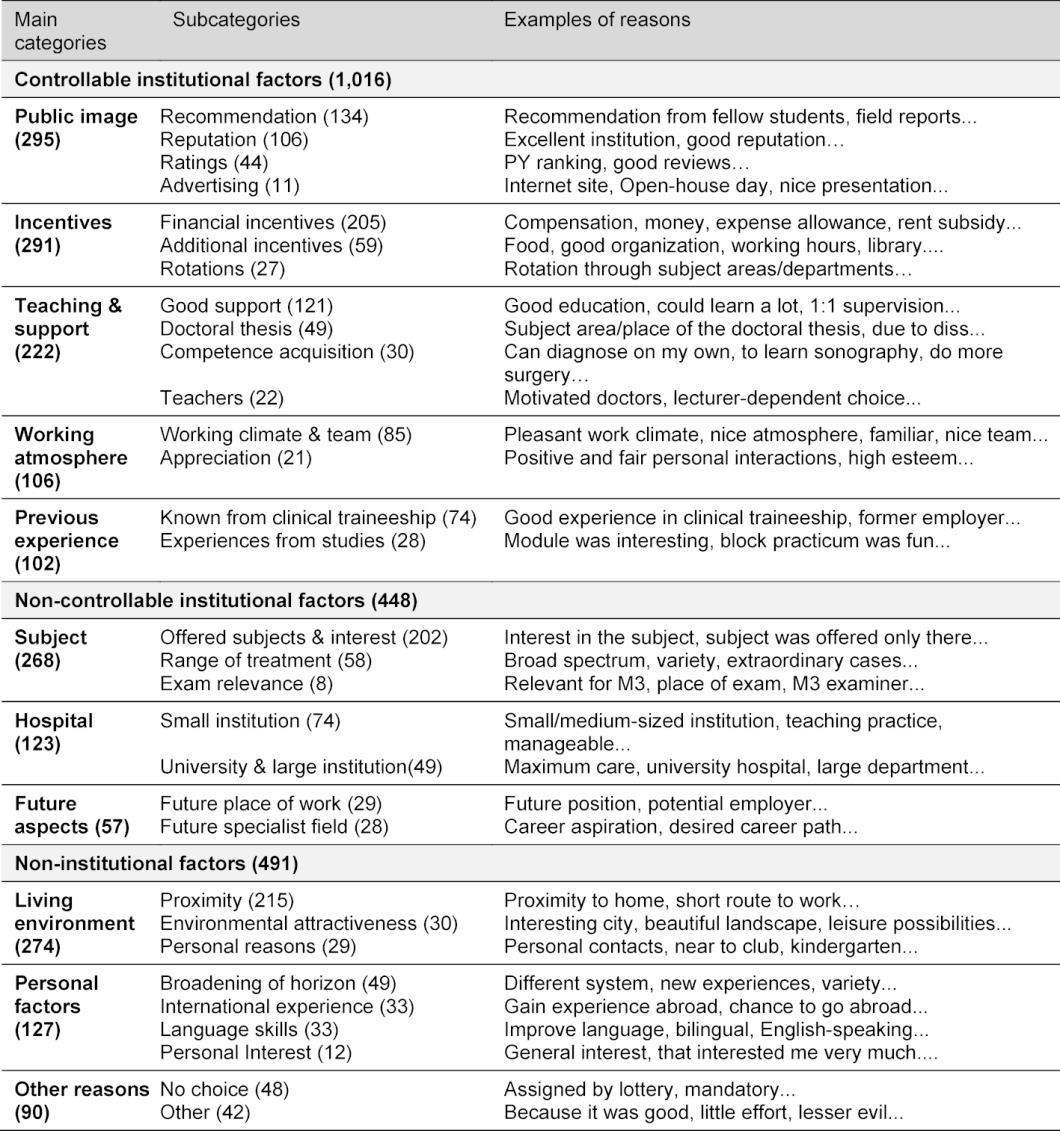
Reasons given for the choice of location

**Table 4:**
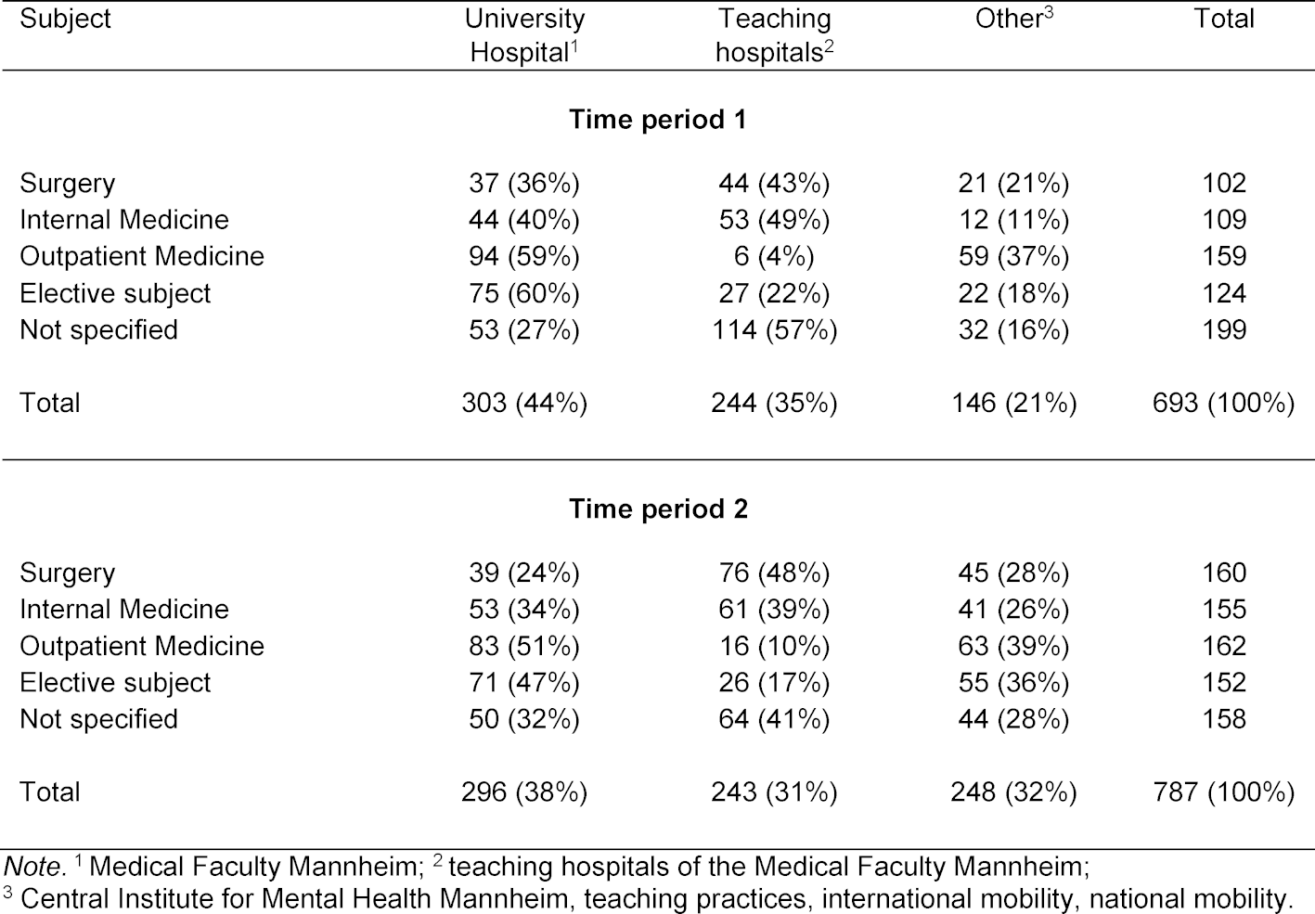
Choice of location for each subject
